# Calycosin and Formononetin Induce Endothelium-Dependent Vasodilation by the Activation of Large-Conductance Ca^2+^-Activated K^+^ Channels (BK_Ca_)

**DOI:** 10.1155/2016/5272531

**Published:** 2016-11-23

**Authors:** Hisa Hui Ling Tseng, Chi Teng Vong, George Pak-Heng Leung, Sai Wang Seto, Yiu Wa Kwan, Simon Ming-Yuen Lee, Maggie Pui Man Hoi

**Affiliations:** ^1^State Key Laboratory of Quality Research in Chinese Medicine, Institute of Chinese Medical Sciences, University of Macau, Avenida da Universidade, Taipa, Macau; ^2^Department of Pharmacology and Pharmacy, Li Ka Shing Faculty of Medicine, University of Hong Kong, Hong Kong; ^3^National Institute of Complementary Medicine, Western Sydney University, Penrith, NSW, Australia; ^4^School of Biomedical Sciences, Faculty of Medicine, The Chinese University of Hong Kong, Shatin, New Territories, Hong Kong

## Abstract

Calycosin and formononetin are two structurally similar isoflavonoids that have been shown to induce vasodilation in aorta and conduit arteries, but study of their actions on endothelial functions is lacking. Here, we demonstrated that both isoflavonoids relaxed rat mesenteric resistance arteries in a concentration-dependent manner, which was reduced by endothelial disruption and nitric oxide synthase (NOS) inhibition, indicating the involvement of both endothelium and vascular smooth muscle. In addition, the endothelium-dependent vasodilation, but not the endothelium-independent vasodilation, was blocked by BK_Ca_ inhibitor iberiotoxin (IbTX). Using human umbilical vein endothelial cells (HUVECs) as a model, we showed calycosin and formononetin induced dose-dependent outwardly rectifying K^+^ currents using whole cell patch clamp. These currents were blocked by tetraethylammonium chloride (TEACl), charybdotoxin (ChTX), or IbTX, but not apamin. We further demonstrated that both isoflavonoids significantly increased nitric oxide (NO) production and upregulated the activities and expressions of endothelial NOS (eNOS) and neuronal NOS (nNOS). These results suggested that calycosin and formononetin act as endothelial BK_Ca_ activators for mediating endothelium-dependent vasodilation through enhancing endothelium hyperpolarization and NO production. Since activation of BK_Ca_ plays a role in improving behavioral and cognitive disorders, we suggested that these two isoflavonoids could provide beneficial effects to cognitive disorders through vascular regulation.

## 1. Introduction

**Figure 1:**
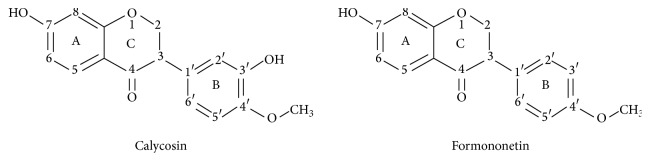
Chemical structures of calycosin and formononetin.

Calycosin and formononetin (Figure 1) are two structurally similar isoflavonoids that are present abundantly in traditional Chinese medicine (TCM) such as Radix Astragali (Huang Qi) and phytoestrogenic herb including* Trifolium pretense *L. (red clover), and they have a long clinical history in treating various cardiovascular diseases [1, 2]. Previous studies have shown that calycosin and formononetin produced antihypertensive effects and improved endothelial and cardiovascular functions [3–6]. They have been shown to display vasoactive effects in various vascular beds [3, 5–7]. In rat aorta, calycosin induced vasodilation mainly by inhibiting voltage-dependent Ca^2+^ channel (VDCC) in vascular smooth muscle [7], while formononetin caused vasodilation by releasing nitric oxide (NO) in endothelial cells, as well as by the activation of large-conductance Ca^2+^-activated K^+^ (BK_Ca_) and ATP-sensitive potassium (K_ATP_) channels in aortic smooth muscle cells [6]. In addition, these two isoflavonoids were reported to ameliorate cerebral ischemia and reperfusion injury by improving endothelial dysfunction [8, 9]. These observation led us to investigate the pharmacological underlying mechanisms of calycosin and formononetin in small resistance arteries (internal diameter ≤ 300 *μ*m) and vascular endothelial cells.

Small resistance arteries are major sites of peripheral vascular resistance and are closely related to endothelial dysfunction and the pathogenesis of hypertension [10, 11]. Interestingly, it has been shown that endothelium-dependent hyperpolarization (EDH) was more pronounced in small resistance arteries than large conduit arteries such as aorta [10, 12]. In the vascular walls, calcium-activated potassium (K_Ca_) channel is the main contributor for endothelium-derived hyperpolarizing factor- (EDHF-) mediated responses, which plays a crucial role in the regulation of vascular tone and the maintenance of systemic blood pressure [13, 14]. Recently, endothelial K_Ca_ channels have been used as new drug targets for cardiovascular diseases such as hypertension to stimulate EDHF and NO production to improve endothelial dysfunction [13, 15–17]. There are three types of K_Ca_ channels based on their conductances, including IK_Ca_ (intermediate conductance), SK_Ca_ (small conductance), and BK_Ca_ (large conductance) [18]. Although IK_Ca_ and SK_Ca_ are the major K_Ca_ channels present in the endothelial cells of arteries, BK_Ca_ channels have been identified in the endothelium of rat pulmonary and mesenteric arteries [19, 20] and cultured endothelial cells [21, 22]. It has been suggested that BK_Ca_ channel has a compensatory role for improving vasoreactivity in environment such as hypertension and cardiovascular diseases [23, 24]. In addition, it was shown that BK_Ca_ channel acts as a negative feedback mechanism for vascular dysfunction impaired by reactive oxygen species (ROS) and is overexpressed in diseases associated with endothelial dysfunction [25–27].

In the present study, we investigated the effects of calycosin and formononetin in rat mesenteric resistance arteries and their underlying mechanisms with a focus on endothelial K^+^ channel. We demonstrated that calycosin and formononetin induced endothelium-dependent vasodilation through NO production and BK_Ca_ channel activation. We also showed that calycosin and formononetin increased NO production through endothelial nitric oxide synthase (eNOS) and neuronal nitric oxide synthase (nNOS) pathway and activated endothelial BK_Ca_ channels in human umbilical endothelial cells (HUVEC). Taken together, our study demonstrated that calycosin and formononetin are endothelial BK_Ca_ channel activators, suggesting a novel mechanism for vasodilation by these isoflavones, and they might be potential for treating vascular and cerebrovascular diseases associated with endothelial dysfunction.

## 2. Material and Methods

### 2.1. Chemicals and Reagents

Calycosin and formononetin were purchased from Shanghai Forever Biotech (Shanghai, China), and they were dissolved in dimethyl sulphoxide (DMSO). All the chemicals were purchased from Sigma-Aldrich (St. Louis, MO, USA), while the cell culture reagents were from Gibco (Carlsbad, CA, USA). All the antibodies used for immunoblotting were purchased from Cell Signaling Technology (Danvers, MA, USA).

### 2.2. Animals

All the procedures were carried out according to the ethical guidelines of the Institute of Chinese Medical Sciences (ICMS), University of Macau, and NIH guide for the Care and Use of Laboratory Animals. Male Sprague-Dawley rats (250 ± 350 g) were killed by cervical dislocation.

### 2.3. Preparation of Resistance Mesenteric Arteries

The mesenteric arterial beds were dissected from rats immediately after cervical dislocation and were kept in cold Krebs–Henseleit buffer with the following compositions (mM): NaCl, 118; KCl, 4.7; MgSO_4_, 1.2; KH_2_PO_4_ 1.2; NaHCO_3_, 25; CaCl_2_, 2.5; D-glucose, 5.5. The Krebs–Henseleit buffer was continuously gassed with a mixture of 95% O_2_/5% CO_2_ to maintain a pH of 7.4. Small (the third branches of the mesenteric artery) mesenteric arteries were placed in a 4-chamber wire myograph (DMT, Tissue Bath System 700MO, Aarhus, Denmark) and were maintained at 37°C in Krebs–Henseleit buffer. The normalized protocol was used as described previously [28]. The tension was recorded by the PowerLab recording system (ADInstruments, Hastings, UK). In endothelium-denuded experiments, the endothelium was removed by rubbing the inner surface of the segment with human hair. In endothelium-intact experiments, the presence of a functional endothelium was examined by precontracting the arteries with methoxamine (10 *μ*M) and then was relaxed by carbachol (10 *μ*M), and this vasodilation was greater than 80%.

### 2.4. Myograph Experimental Protocol

After 30 min equilibration, arteries were firstly precontracted by methoxamine (10 *μ*M). Once the tension was stable, calycosin (1 nM–100 *μ*M) and formononetin (1 nM–100 *μ*M) were added cumulatively to produce a concentration-dependent response in endothelium-intact and endothelium-denuded mesenteric arteries. The effects of vasodilation in response to calycosin and formononetin were also examined with preincubation of indomethacin, N_*ω*_-nitro-l-arginine methyl ester (L-NAME), tetraethylammonium chloride (TEACl), apamin, charybdotoxin (ChTX), iberiotoxin (IbTX), and glibenclamide for 30 min, before precontracting with methoxamine. In some experiments, the arteries were precontracted with high K^+^ (60 mM KCl) Krebs–Henseleit buffer by replacing NaCl with KCl in the standard Krebs–Henseleit solution, as described previously [28].

### 2.5. Cell Culture

Human umbilical vein endothelial cells (HUVECs) were purchased from Life Technologies (Carlsbad, CA, USA) and were cultured in Hams F-12k nutrient medium supplemented with 15% fetal bovine serum, 1% penicillin-streptomycin, 100 mg/mL heparin, and 10 mg/mL endothelial cell growth supplement (Sigma-Aldrich, St. Louis, MO, USA) at 37°C under an atmosphere of 5% CO_2_ in air. The cells were used in passages 2–6. Plated cells were allowed to adhere for 2 h before patch clamp experiments. For the experiments measuring NO production, and the expressions of different nitric oxide synthases (NOS), calycosin and formononetin were treated for 1 h in HUVEC.

### 2.6. NO Production Assay

NO was measured by a Nitric Oxide Assay Kit (Abcam, Cambridge, UK) following the manufacturer's instruction. Briefly, 75 *μ*L of cell supernatants was mixed with 5 *μ*L enzyme cofactor solution and 5 *μ*L nitrate reductase. Following 2 h of incubation for converting nitrates to nitrites, 5 *μ*L enhancer was added to each sample and incubated for 30 min. 5 *μ*L DAN Probe was then added and incubated for 10 min. After that, 5 *μ*L NaOH was added in the mixture for 10 min. The fluorescence intensity was detected by a microplate reader (SpectaMax M5, Molecular Devices, USA) using excitation at 360 nm and emission at 450 nm wavelengths.

### 2.7. Patch Clamp Experiments

All the cells were superfused with the extracellular solution with the following compositions (mM): NaCl, 140; KCl, 5.4; CaCl_2_, 1.8; MgCl_2_, 1; NaH_2_PO_4_ 0.33; HEPES, 5; D-glucose, 5.5; pH 7.4. The recordings were made by using an Axopatch-200B amplifier, Digidata-1321 interface, and pClamp10.0 software (Axon Instruments, Forster City, CA). In the outward K^+^ channel experiment, the pipettes (resistance 2-3 MΩ) were filled with intracellular pipette solution (mM): KCl, 140; MgCl_2_, 2.5; D-glucose, 10; HEPES, 5; pH 7.2. In some experiments, [Ca^2+^]_i_ was adjusted to 250, 500, or 1000 nM by administrating 6.7, 8.0, or 8.9 mM CaCl_2_ to the intracellular pipette solution, respectively, and in the presence of 10 mM EGTA. The cells were clamped at a holding potential of −60 mV and various potential steps from −100 mV to +100 mV with 10 mV increments, and the currents were stimulated by a series of 250 ms.

### 2.8. [Ca^2+^]_i_ Measurements

The intracellular Ca^2+^ concentration ([Ca^2+^]_i_) was measured in single cells as previously described [29]. Cells were loaded with Fluo-4 AM (2 *μ*M, Molecular Probes, US) in Tyrode solution containing 136.5 mM NaCl, 5.4 mM KCl, 0.53 mM MgCl_2_, 1.8 mM CaCl_2_, 0.33 mM NaH_2_PO_4_, 5.5 mM glucose, and 5.5 mM HEPES (pH 7.4, adjusted with NaOH) for 30 min at 37°C. Fluo-4 fluorescence intensity (494 nm excitation; 506 nm emission) was sampled at 5 s intervals using a Cell^R^ system (MT20, Olympus, US).

### 2.9. Western Blot Analysis

After indicated treatment, the protein was extracted with ice-cold lysis buffer, and the concentrations of lysates were measured by the bicinchoninic acid kit (Pierce, US). 30 *μ*g proteins were used and separated by 10% SDS-PAGE gels and were transferred onto the nitrocellulose membranes. Membranes were incubated with primary antibodies (eNOS, p-eNOS, nNOS, and iNOS antibody using 1/1000 dilution, whereas GAPDH antibody using 1/4000 dilution) overnight at 4°C, and secondary antibodies (anti-rabbit with 1/1000 dilution) for 1 hr, and blots were developed by enhanced chemiluminescence (GE Healthcare Life Sciences, UK) with an imaging system (Bio-Rad Laboratories, USA). GAPDH were used as housekeeping controls.

### 2.10. Data and Statistical Analysis

Data were expressed as mean ± SEM. vasodilation responses in each segment were expressed as a percentage of relaxation. The dose-response curves were fitted to a logistic equation described previously [30]. The maximum percentage of relaxation (*R*_max_) and the concentration required to produce 50% of maximal response tone (EC_50_) were calculated from the fitted curves. Currents curves were fitted by Boltzmann equations. The curve fitting and statistical analyzes were determined using GraphPad Prism 5 (San Diego, CA, USA). Significant differences were analyzed by *t*-test, one-way ANOVA followed by a Dunnett's test, or two-way ANOVA followed by a Bonferroni* post hoc* test. *P* < 0.05 was considered as significant.

## 3. Results

### 3.1. Effects of Endothelial Removal, L-NAME, and K^+^ Channel Inhibitors on Vasodilation in Response to Calycosin

**Figure 2:**
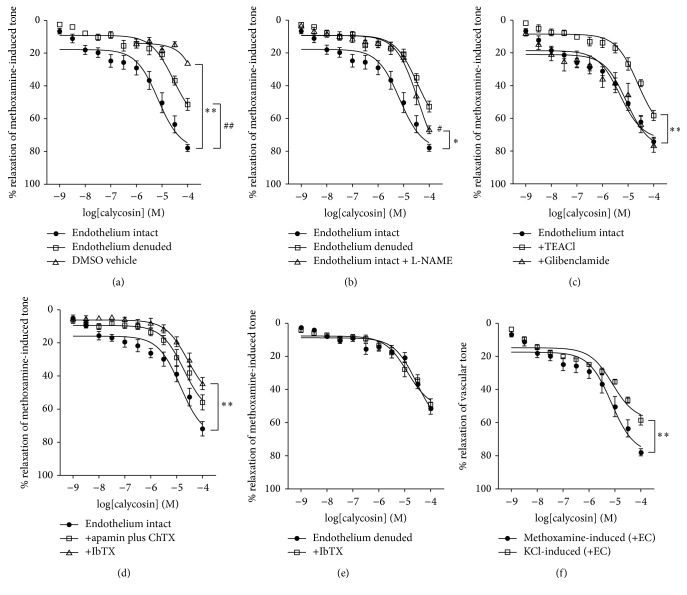
Concentration-response curves for calycosin-induced relaxation in the rat mesenteric arteries. (a) Vasorelaxation induced by calycosin with DMSO vehicle, or in the presence and absence of endothelium (^*∗∗*^ *P* < 0.01; ^##^ *P* < 0.01). (b) Calycosin-induced vasorelaxation in the presence and absence of endothelium, or L-NAME (300 *μ*M) pretreatment in endothelium-intact arteries (^*∗*^ *P* < 0.05; ^#^ *P* < 0.05 versus endothelium denuded). (c, d) Calycosin-induced vasorelaxation with pretreatments of either TEACl (3 mM; ^*∗∗*^ *P* < 0.01), glibenclamide (10 *μ*M), apamin (50 nM) plus ChTX (50 nM), or IbTX (200 nM; ^*∗∗*^ *P* < 0.01). (e) Calycosin-induced vasorelaxation with IbTX (200 nM) pretreatment in endothelium-denuded arteries. (f) Calycosin-induced vasorelaxation with precontractions by methoxamine (10 *μ*M) or KCl (60 mM; ^*∗∗*^ *P* < 0.01) in endothelium-intact arteries. Data were shown as mean ± SEM. ChTX, charybdotoxin; IbTX, iberiotoxin; TEACl, tetraethylammonium chloride.

Calycosin induced dose-dependent vasodilation with methoxamine (10 *μ*M) precontraction in endothelium-intact small mesenteric arteries (Figure 2(a), EC_50_ = 7.7 ± 0.05 *μ*M, *R*_max_ = 87.9 ± 2.0%; *n* = 6). The removal of endothelium significantly reduced this effect (Figure 2(a), EC_50_ = 28.6 ± 0.12 *μ*M, *R*_max_ = 51.3 ± 4.5%; *n* = 6). Next, we examined whether endothelium-derived NO was involved in this vasodilation. Preincubation with L-NAME (300 *μ*M), a nitric oxide synthase inhibitor, partially reduced calycosin-induced vasodilation (Figure 2(b), EC_50_ = 40.4 ± 0.08 *μ*M, *R*_max_ = 75.6 ± 3.4%; *n* = 5). Notably, as shown in Figure 2(b), the inhibitory effects of endothelium denudation and L-NAME preincubation on calycosin-induced vasodilation were similar. However, indomethacin (10 *μ*M), cyclooxygenase inhibitor, did not affect the vasodilation by calycosin (data not shown).

Next, we examined whether K^+^ channels were also involved in calycosin-induced vasodilation. With pretreatment of TEACl (3 mM), a nonspecific inhibitor of K^+^ channels, the vasodilation effect was significantly reduced when compared to control (Figure 2(c), EC_50_ = 25.1 ± 0.12 *μ*M, *R*_max_ = 58.3 ± 5.1%; *n* = 6). Similarly, pretreatment with BK_Ca_ channel inhibitor, IbTX (200 nM), significantly reduced calycosin-induced vasodilation (Figure 2(d), EC_50_ = 28.3 ± 0.08 *μ*M, *R*_max_ = 44.5 ± 3.9%; *n* = 6). However, with pretreatment of K_Ca_ channel inhibitors, apamin (50 nM) plus ChTX (50 nM), the vasodilation was reduced to a smaller extent (Figure 2(d), EC_50_ = 20.7 ± 0.12 *μ*M, *R*_max_ = 56.0 ± 4.3%; *n* = 6). Conversely, glibenclamide (10 *μ*M), a K_ATP_ channel inhibitor, had no effect on calycosin-induced vasodilation (Figure 2(c), EC_50_ = 8.9 ± 0.08 *μ*M, *R*_max_ = 76.4 ± 6.2%; *n* = 6). Surprisingly, pretreatment with IbTX (200 nM) in endothelium-denuded arteries had no effect on calycosin-induced vasodilation (Figure 2(e)). Furthermore, calycosin-induced vasodilation was reduced with KCl (60 mM) precontraction compared to methoxamine precontraction in endothelium-intact arteries (Figure 2(f), EC_50_ = 8.06 ± 0.08 *μ*M, *R*_max_ = 58.5 ± 4.8%; *n* = 5). These data showed that calycosin induced vasorelaxation via both endothelium-dependent and endothelium-independent pathways. More interestingly, the data also suggested that BK_Ca_ channels are closely related to the endothelium-dependent vasorelaxation.

### 3.2. Effects of Endothelial Removal, L-NAME, and K^+^ Channel Inhibitors on Vasodilation in Response to Formononetin

**Figure 3:**
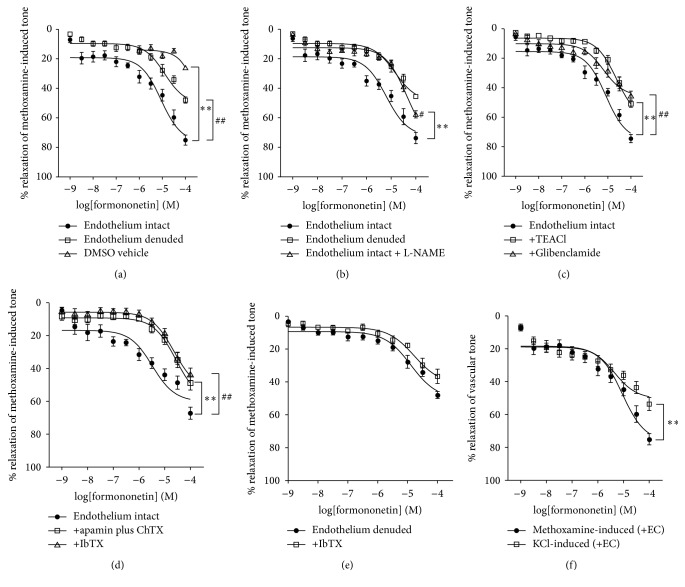
Concentration–response curves for formononetin-induced relaxation in the rat mesenteric arteries. (a) Formononetin-induced vasorelaxation with DMSO vehicle, or in the presence and absence of endothelium (^*∗∗*^ *P* < 0.01; ^##^ *P* < 0.01). (b) Formononetin-induced vasorelaxation in the presence and absence of endothelium, or L-NAME (300 *μ*M) preincubation in endothelium-intact arteries (^*∗∗*^ *P* < 0.01; ^#^ *P* < 0.05 versus endothelium denuded). (c, d) Formononetin-induced vasorelaxation with pretreatments of either TEACl (3 mM; ^*∗∗*^ *P* < 0.01), glibenclamide (10 *μ*M; ^##^ *P* < 0.01), apamin (50 nM) plus ChTX (50 nM; ^*∗∗*^ *P* < 0.01), or IbTX (200 nM; ^##^ *P* < 0.01). (e) Formononetin-induced vasorelaxation with IbTX (200 nM) preincubation in endothelium-denuded arteries. (f) Formononetin-induced vasorelaxation with precontractions of methoxamine (10 *μ*M) or KCl in endothelium-intact arteries (60 mM; ^*∗∗*^ *P* < 0.01). Data were shown as mean ± SEM. ChTX, charybdotoxin; IbTX, iberiotoxin; TEACl, tetraethylammonium chloride.

Formononetin also induced concentration-dependent vasodilation after methoxamine (10 *μ*M) precontraction (Figure 3(a), EC_50_ = 9.6 ± 0.12 *μ*M, *R*_max_ = 79.9 ± 3.4%, *n* = 6), and removal of the endothelium (Figure 3(a), EC_50_ = 13.9 ± 0.09 *μ*M, *R*_max_ = 63.6 ± 3.2%, *n* = 6) or preincubation with L-NAME (300 *μ*M) (Figure 3(b), EC_50_ = 42.8 ± 0.30 *μ*M, *R*_max_ = 56.3 ± 4.5%, *n* = 5) significantly reduced this effect. On the other hand, indomethacin (10 *μ*M) did not affect this vasodilation (data not shown).

As shown in Figure 3(c), formononetin-induced vasodilation was significantly inhibited with pretreatments of TEACl (3 mM, EC_50_ = 24.3 ± 0.20 *μ*M, *R*_max_ = 50.8 ± 4.6%; *n* = 6) or glibenclamide (10 *μ*M, EC_50_ = 6.67 ± 0.12 *μ*M, *R*_max_ = 45.4 ± 3.9%; *n* = 7). The vasodilation effect by formononetin was also reduced with pretreatment of IbTX (200 nM), or the combination of apamin plus ChTX (both 50 nM, Figure 3(d), IbTX: EC_50_ = 23.7 ± 0.18 *μ*M, *R*_max_ = 43.6 ± 4.2%; *n* = 6; *P* < 0.01; A+C: EC_50_ = 27.8 ± 0.17 *μ*M, *R*_max_ = 48.9 ± 4.0%; *n* = 6). In addition, pretreatment with IbTX (200 nM) did not affect formononetin-induced vasodilation in endothelium-denuded arteries (Figure 3(e)). Formononetin-induced vasodilation was significantly reduced with KCl (60 mM) precontraction compared to methoxamine precontraction in endothelium-intact arteries (Figure 3(f), EC_50_ = 4.38 ± 0.05 *μ*M, *R*_max_ = 53.5 ± 3.9%, *n* = 5). Similar to the effects of calycosin, these data showed that formononetin induced vasorelaxation via both endothelium-dependent and endothelium-independent pathways. More interestingly, the data also suggested that BK_Ca_ channels are closely related to the endothelium-dependent vasorelaxation.

### 3.3. Effect of Calycosin and Formononetin on NO Production and eNOS, iNOS, and nNOS Expression in HUVEC

**Figure 4:**
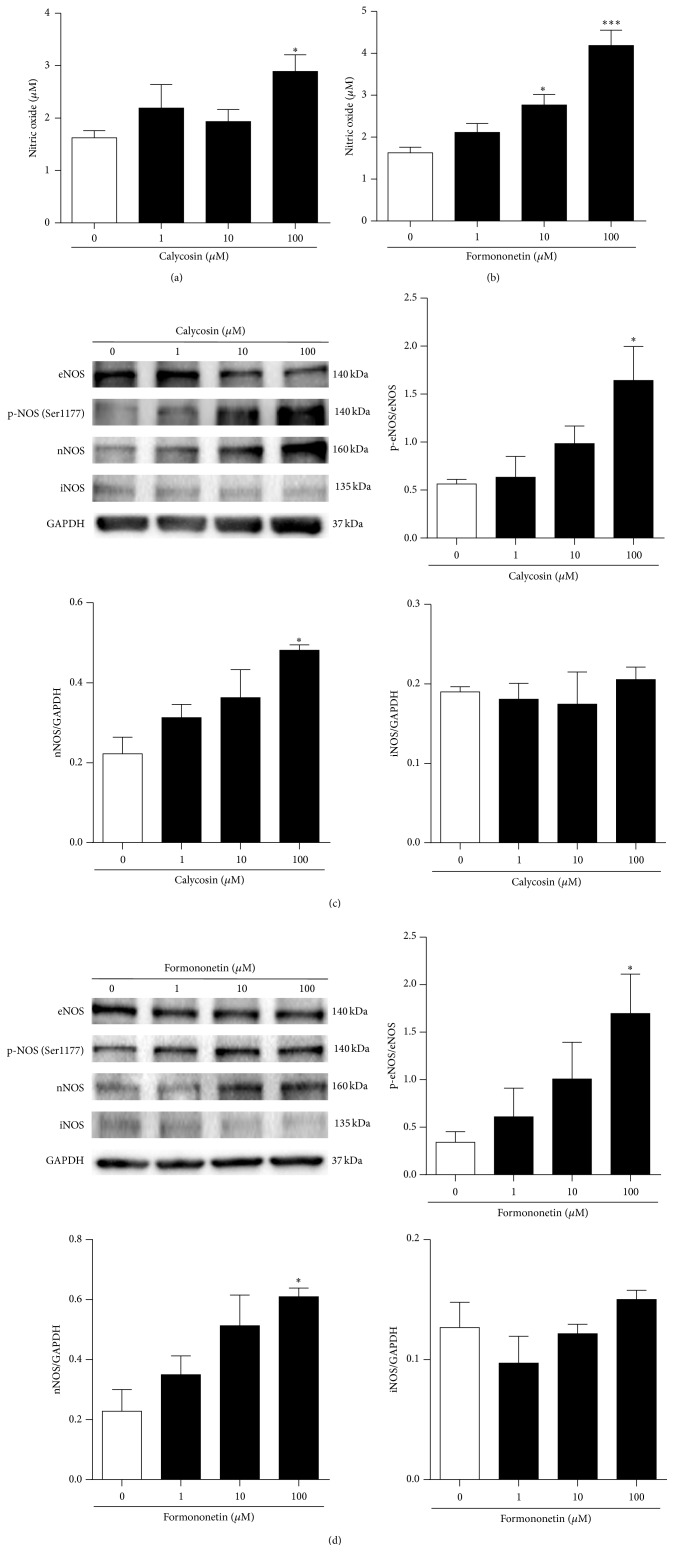
Calycosin and formononetin induced NO production via eNOS and nNOS pathways in HUVEC. (a, b) NO level was determined by a NO assay kit. HUVEC was incubated with DMSO, calycosin (1–100 *μ*M), or formononetin (1–100 *μ*M) for 1 h (*n* = 5). (c, d) Representative immunoblots and graphs for the protein expressions of eNOS, phosphorylation of eNOS, nNOS, iNOS, or GAPDH after (c) calycosin (1–100 *μ*M) or (d) formononetin (1–100 *μ*M) treatment for 1 h (*n* = 3-4). Data were shown as mean ± SEM. ^*∗*^ *P* < 0.05, ^*∗∗∗*^ *P* < 0.001 versus untreated cells.

In order to further determine whether these two isoflavonoids could regulate the production of NO and the expression of NOS in endothelial cells, HUVEC was employed as a cellular model. HUVEC is a widely* in vitro* cell model for the study of the regulation of endothelial function and the vascular diseases [31].  Figure 4(a) showed that calycosin increased NO production in a dose-dependent manner in HUVEC, and similar effect was also observed with formononetin (Figure 4(b)). There are three isoforms of NOS, including eNOS, inducible nitric oxide synthase (iNOS), and nNOS, which are responsible for the generation of NO in the vascular endothelium [32]. It has been reported that eNOS and nNOS are coexpressed in endothelial cells while iNOS is not [33]. Our results demonstrated that both calycosin (100 *μ*M) and formononetin (100 *μ*M) significantly induced the activation of eNOS (Figures 4(c) and 4(d)). In addition, both isoflavonoids also upregulated nNOS expression (Figures 4(c) and 4(d)). In contrast, iNOS expression was unaffected by neither calycosin nor formononetin (Figures 4(c) and 4(d)). These data suggested that calycosin and formononetin induced endothelium-dependent vasorelaxation via NO production through eNOS and nNOS.

### 3.4. Effect of Calycosin on Outward Currents in HUVEC

**Figure 5:**
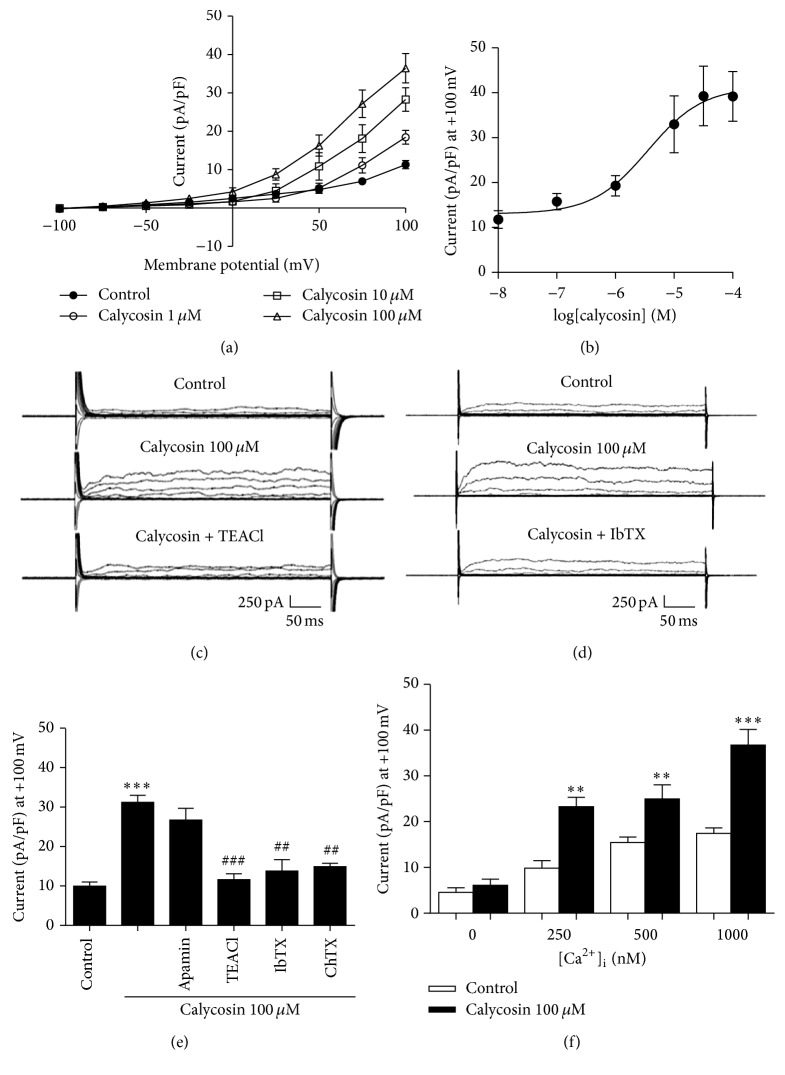
Calycosin increased outward currents in HUVEC through BK_Ca_ channel. (a) Current-voltage (*I*/*V*) relationship in response to calycosin (1–100 *μ*M). (b) Dose-response curve for whole cell recording of currents at +100 mV with different concentrations of calycosin (10 nM–100 *μ*M). (c, d) Representative trace of currents that were recorded in response to calycosin in the absence or presence of (c) TEACl (1 mM) or (d) IbTX (200 nM). (e, f) Whole cell recording of currents at +100 mV in response to calycosin (100 *μ*M) in the presence of (e) apamin (200 nM), IbTX (200 nM), ChTX (200 nM), or TEACl (1 mM; ^*∗∗∗*^ *P* < 0.001 versus control; ^##^ *P* < 0.01 and ^###^ *P* < 0.001 versus calycosin-treated cells), or (f) with different [Ca^2+^]_i_ as indicated (^*∗∗*^ *P* < 0.01 and ^*∗∗∗*^ *P* < 0.001 versus calycosin-treated cells with free [Ca^2+^]_i_). Data were shown as mean ± SEM. ChTX, charybdotoxin; IbTX, iberiotoxin; TEACl, tetraethylammonium chloride.

The activation of K_Ca_ is the major mechanism for EDH. We observed that exposure of calycosin induced dose-dependent outward currents in HUVEC recorded by whole cell patch clamp. The whole cell currents of HUVEC were recorded with 250 ms voltage steps between −100 mV and +100 mV from a holding potential of −60 mV. As shown in Figures 5(a) and 5(b), calycosin (10 nM–100 *μ*M) increased dose-dependent outward currents in HUVEC (*n* = 9). At 100 *μ*M calycosin, the current at +100 mV was significantly increased (Figures 5(a) and 5(c), 31.2 ± 1.7 pA/pF; *n* = 7) compared to control (9.9 ± 1.0 pA/pF), and this current was abolished by TEACl, a nonspecific inhibitor of K^+^ channels (1 mM, Figures 5(c) and 5(e); 11.6 ± 1.5 pA/pF; *n* = 6).

Although IK_Ca_ and SK_Ca_ are the major K_Ca_ channels present in the endothelial cells, BK_Ca_ is also expressed moderately, and it was identified in HUVEC [22, 23]. We observed that K_Ca_ channels in calycosin induced outward currents in HUVEC. An IK_Ca_ channel inhibitor, apamin (100 nM), had no effect on the outward currents induced by calycosin (Figure 5(e), *n* = 6). However, it was abolished by BK_Ca_ inhibitors, IbTX (200 nM, Figures 5(d) and 5(e); 13.8 ± 2.8 pA/pF; *n* = 6) and ChTX (200 nM, Figure 5(e); 14.9 ± 0.9 pA/pF; *n* = 7). In addition, by maintaining [Ca^2+^]_i_ at 250 nM, 500 nM, or 1000 nM, calycosin (100 *μ*M) significantly increased the outward currents stimulated by single +100 mV step, compared with Ca^2+^-free solution containing 10 mM EGTA (Figure 5(f), *n* = 7–9). These data strongly suggested that calycosin mainly activated endothelial BK_Ca_ channels but has minimal effects on IK_Ca_ or SK_Ca_.

### 3.5. Effect of Formononetin on Outward Currents in HUVEC

**Figure 6:**
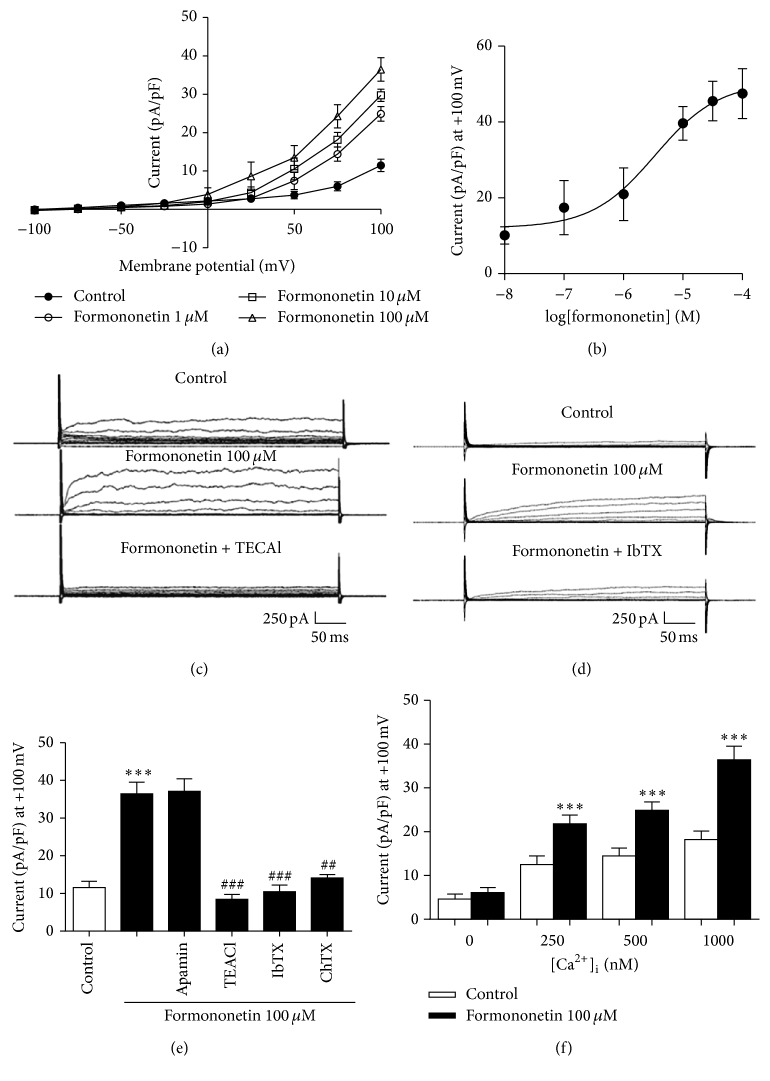
Formononetin increased outward currents in HUVEC through BK_Ca_ channel. (a) *I*/*V* graph in response to formononetin (1–100 *μ*M). (b) Dose-response curve for whole cell recording of currents at +100 mV with different concentrations of formononetin (10 nM–100 *μ*M). (c, d) Representative trace of currents that were recorded in response to formononetin, in the absence and presence of (c) TEACl (1 mM) or (d) IbTX (200 nM). (e, f) Whole cell recording of currents at +100 mV in response to formononetin (100 *μ*M) in the presence of (e) apamin (200 nM), IbTX (200 nM), ChTX (200 nM), or TEACl (1 mM; ^*∗∗∗*^ *P* < 0.001 versus control; ^##^ *P* < 0.01 and ^###^ *P* < 0.001 versus formononetin-treated cells), or (f) with different [Ca^2+^]_i_ as indicated (^*∗∗∗*^ *P* < 0.001 versus formononetin-treated cells with free [Ca^2+^]_i_). Data were shown as mean ± SEM. ChTX, charybdotoxin; IbTX, iberiotoxin; TEACl, tetraethylammonium chloride.

It was observed that formononetin (10 nM–100 *μ*M) significantly increased the outward currents in a concentration-dependent manner in HUVEC, similar to the effects of calycosin (Figures 6(a) and 6(b), *n* = 6-7). At 100 *μ*M, formononetin significantly increased the outward currents compared to control at +100 mV step (Figure 6(a), control: 1.4 ± 1.6 pA/pF, formononetin: 36.4 ± 3.1 pA/pF; *n* = 6). This current was abolished by TEACl (1 mM, Figures 6(c), and 6(e); 15.7 ± 3.6 pA/pF; *n* = 6), or IbTX (200 nM, Figures 6(d) and 6(e), 14.1 ± 0.9 pA/pF; *n* = 5), or ChTX (200 nM, Figure 6(e), 14.1 ± 0.9 pA/pF; *n* = 5). However, apamin (100 nM, Figure 6(e), 37.1 ± 3.2 pA/pF; *n* = 6) had no effect on these outward currents. Maintaining [Ca^2+^]_i_ at 250 nM, 500 nM, or 1000 nM, formononetin (100 *μ*M) significantly increased the outward currents at +100 mV compared with Ca^2+^-free solution containing 10 mM EGTA (Figure 6(f), *n* = 6). These data suggested that formononetin mainly activated endothelial BK_Ca_ channels but has minimal effects on IK_Ca_ or SK_Ca_.

### 3.6. Effect of Calycosin and Formononetin on [Ca^2+^]_i_ in HUVEC

**Figure 7:**
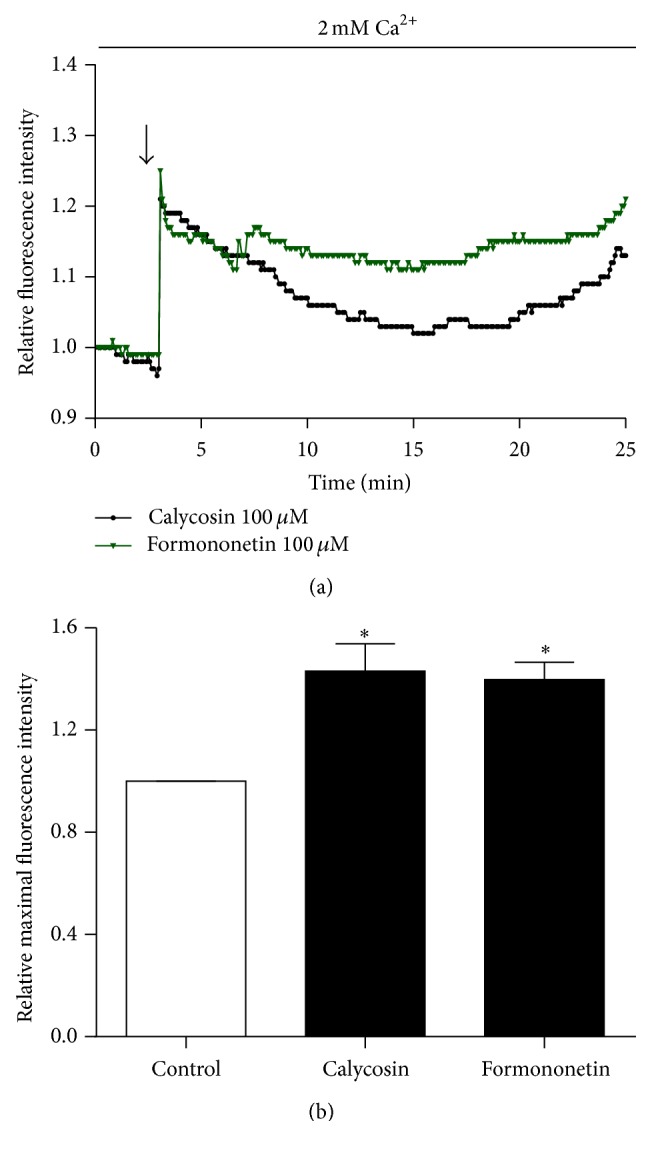
Calycosin and formononetin-induced Ca^2+^ response in HUVEC. (a, b) HUVEC was loaded with Fluo-4 in Tyrode solution containing 2 mM Ca^2+^. Representative graph and relative fluorescence intensity in intracellular Ca^2+^ concentration ([Ca^2+^]_i_), evoked by calycosin (100 *μ*M) and formononetin (100 *μ*M) over the time course (*n* = 3). Data were shown as mean ± SEM. ^*∗*^ *P* < 0.05 versus control.

Endothelial [Ca^2+^]_i_ elevation has been implicated in endothelium-mediated vasodilation [18] and is also needed for K_Ca_ activation [34]. Since calycosin and formononetin increased outward currents via endothelial K_Ca_ channel, we next examined their effects on [Ca^2+^]_i_ in HUVEC. As expected, we observed a rapid increase in [Ca^2+^]_i_ in response to calycosin (Figures 7(a) and 7(b)). Similar results also showed that formononetin evoked Ca^2+^ influx (Figures 7(a) and 7(b)). Both of these two drugs induced approximately 45% increases in [Ca^2+^]_i_ compared to untreated cells. Therefore, these data suggested that calycosin and formononetin activated endothelial BK_Ca_ channels probably by increasing [Ca^2+^]_i_.

## 4. Discussion

In the present study, we investigated the endothelial beneficial effects of calycosin and formononetin, two isoflavonoids isolated from the well-known antihypertensive herb, Radix Astragali, in rat small resistance arteries and HUVEC. The chemical structures of calycosin and formononetin are very similar, with an extra hydroxyl group at C-3 position of the B-ring in calycosin [35]. Both isoflavonoids were shown to provide beneficial effects in vascular tone regulation and improvement in endothelial and cardiovascular dysfunction [3–6].

Here, we demonstrated that calycosin and formononetin produced very similar effects in rat small mesenteric arteries, that is, inducing vasorelaxation through endothelium-dependent and endothelium-independent pathways. We observed that relaxations elicited by both isoflavonoids could be reduced by endothelial disruption and were sensitive to L-NAME (inhibitor of NOS), apamin plus charybdotoxin (inhibitors of SK_Ca_ and IK_Ca_), and iberiotoxin (IbTX) (inhibitor of BK_Ca_). Notably, the sensitivity to IbTX was only observed in endothelium-intact, but not in endothelium-denuded vessels, indicating the involvement of BK_Ca_ channels present in the endothelium. The relaxation elicited by both isoflavonoids was also reduced (to a lesser extent) when the arteries were contracted with high KCl (60 mM) depolarizing solution, indicating the inhibition of VDCC was also partly involved. Pretreatment with indomethacin to inhibit prostaglandin production did not affect the vasodilation, indicating that PGI_2_ was not involved. Although calycosin and formononetin are very similar in structure, differential properties of their effects were also observed. Our data showed that relaxation induced by formononetin was sensitive to glibenclamide (K_ATP_ channel inhibitor), but not calycosin-induced relaxation. Besides, we also observed some discrepancies in the effects of calycosin and formononetin in blood vessels from various vascular beds. Previous study intact aortic rings reported that the relaxation elicited by calycosin was endothelium-independent by acting as Ca^2+^ channel blocker [7]. However, here in rat mesenteric arteries we observed that calycosin-elicited relaxation is both endothelium-dependent and endothelium-independent. For formononetin, results from previous studies in rat aortic rings showed that formononetin elicited relaxation through endothelium-dependent pathway involving NO synthesis, and endothelium-independent involving iberiotoxin- (IbTX-) sensitive BK_Ca_ channel, glibenclamide-sensitive K_ATP_ channel, and the inhibition of VDCC [5, 6, 36]. Here in rat mesenteric arteries, we observed very similar effects of formononetin, but in our preparation the sensitivity to IbTX was only observed in endothelium-intact vessels, indicating a more important role of endothelial BK_Ca_ channels in the mesenteric resistance arteries. The discrepancies observed for calycosin and formononetin in rat aorta and mesenteric arteries may be explained by the differential physiological characteristics exhibited by conduit and resistance arteries for vascular homeostasis. In fact, it has been recognized that endothelium-dependent vasoactivities are more pronounced in small resistance arteries than in large conduit arteries such as aorta, and small resistance arteries are closely related to endothelial dysfunction [10, 12]. Thus, our results suggested that calycosin and formononetin could effectively promote endothelial functions in the small resistance arteries, and endothelial BK_Ca_ channels might have an important role.

BK_Ca_ channels are mainly expressed in vascular smooth muscle cells, but recent studies demonstrated that BK_Ca_ channels are also present in vascular endothelium or freshly isolated endothelial cells from the small resistance arteries [19, 20]. When expressed in endothelial cells, BK_Ca_ channels are observed to regulate NO and EDH production, and their important roles have been implicated in disease conditions such as ischemic hypoxia [19, 23, 37, 38]. In line with this, we demonstrated that both calycosin and formononetin significantly increased NO production and upregulated the activities and expressions of eNOS and nNOS, without affecting iNOS in HUVEC. It has been suggested that endothelial BK_Ca_ channel might serve as a compensatory mechanism for improving vasoreactivity in disease environments such as hypertension and could be a potential therapeutic target for the regulation of blood pressure and flow through increased NO production vascular hyperpolarization [20, 39].

By using whole cell patch clamp, we further showed that IbTX-sensitive outward-rectifying currents were induced by exposure to calycosin or formononetin in a dose-dependent manner in HUVEC, an endothelial cell model commonly used for endothelial functions. Moreover, these currents were also sensitive to charybdotoxin (also an inhibitor at BK_Ca_ channel), but not apamin (selective SK_Ca_ channel inhibitor). Buffering intracellular Ca^2+^ by EGTA significantly reduced calycosin- and formononetin-induced outward currents, indicating that the activation of the K^+^ channels was dependent on intracellular calcium which further indicated the characteristic of BK_Ca_ channel [40]. We further demonstrated that calycosin and formononetin could induced an [Ca^2+^]_i_ increase in HUVEC, providing more evidences that calycosin and formononetin activate endothelial BK_Ca_ channel by stimulating [Ca^2+^]_i_ increase.

Until recently, the emerging view of cerebrovascular dysregulation was implicated not only in cerebrovascular diseases, such as stroke, but also in neurodegeneration, like Alzheimer's disease (AD) [41]. Particularly, the inhibition of BK_Ca_ activity was observed in 3xTg AD model mice and might be involved in early dysfunction in the AD brain [42]. Several studies suggested that the activation of BK_Ca_ channels might be new therapeutic target for improving behavioral and cognitive disorders [43, 44]. Since our results demonstrated that calycosin and formononetin act as novel BK_Ca_ activators and also played a role in the regulation in small resistance arteries, we postulated these two isoflavonoids might have potential effects on cognitive disorders through the regulation of cerebral microcirculation by activating BK_Ca_ channels.

## 5. Conclusions

In summary, our findings demonstrated that calycosin and formononetin induced vasodilation in rat small mesenteric arteries involving both the endothelium and the vascular smooth muscle. The endothelium-dependent responses were associated with eNOS/nNOS-dependent NO production and endothelium hyperpolarization, possibly by directly activating endothelial BK_Ca_ channels. Therefore, we suggested that these isoflavonoids might provide potential therapeutic regiments to improve endothelial functions for treating diseases related to abnormal vascular alteration, such as hypertension, cardiovascular diseases, and cerebrovascular-circulation related cognitive disorders such as stroke and vascular dementia.

## Abbreviations

BK_Ca_: Large-conductance Ca^2+^-activated K^+^
ChTX: Charybdotoxin
EC_50_: Concentration producing 50% of the maximum effect
EDHF: Endothelium-derived hyperpolarizing factor
Em: Membrane potential
HUVEC: Human umbilical vein endothelial cells
IbTX: Iberiotoxin
K_ATP_: ATP-sensitive potassium channels
K_Ca_: Ca^2+^-activated K^+^
L-NAME: N_*ω*_-nitro-l-arginine methyl ester
NO: Nitric oxide
NOS: Nitric oxide synthase
eNOS: Endothelial nitric oxide synthase
iNOS: Inducible nitric oxide synthase
nNOS: Neuronal nitric oxide synthase
PGI_2_: Prostacyclin
TEACl: Tetraethylammonium chloride
VDCC: Voltage-dependent calcium channel.
